# Case Report: Partial neurectomy and limb-sparing treatment for SOX-10 expressing epithelioid malignant nerve sheath tumour of the tibial nerve

**DOI:** 10.3389/fvets.2026.1773674

**Published:** 2026-04-08

**Authors:** Alexander Spencer-Taylor, Dylan Yaffy, Juan Marti, Maria Miguel-Garcés, Francesca Raimondi

**Affiliations:** 1 Southern Counties Veterinary Specialists, Independent Vetcare (IVC) Evidensia, Ringwood, United Kingdom; 2 Department of Pathobiology and Population Sciences, The Royal Veterinary College, London, United Kingdom

**Keywords:** case report, epithelioid malignant peripheral nerve sheath tumour (EMPNST), neoplasia, SOX10, tibial nerve

## Abstract

**Introduction:**

Epithelioid-variant malignant peripheral nerve sheath tumours (EMPNST) are rare in veterinary medicine and typically have poor outcomes. Limb-sparing partial neurectomy is used for distal peripheral nerve sheath tumours but has not previously been described for epithelioid variants.

**Case description:**

An 8.5-year-old neutered male Dobermann presented with non–weight-bearing left pelvic limb lameness and severe pain on hock flexion. CT and ultrasound showed a homogeneously thickened, contrast-enhancing segment of the left tibial nerve with muscle atrophy. Limb-sparing partial neurectomy with 2-cm margins was performed. Histopathology revealed a poorly demarcated infiltrative malignant neoplasm, completely excised. Immunohistochemistry was strongly positive for Sox-10 and negative for S-100. The dog improved rapidly, and at 12- and 24-month follow-ups showed no recurrence or metastasis.

**Conclusion:**

This case demonstrates an atypical anatomical and histopathological presentation of canine EMPNST and shows that complete limb-sparing partial neurectomy can achieve long-term remission without compromising limb function.

## Introduction

Peripheral nerve sheath tumours (PNSTs) are relatively uncommon neoplasms originating from Schwann cells, fibroblasts, or perineural cells, accounting for approximately 2% of all canine tumours (1). Malignant variants often show poor differentiation, rendering the cell origin unclear in most cases (2). These tumours commonly involve nerves or nerve roots, predominantly in the caudal cervical and cranial thoracic regions, although they have been known to involve cranial nerves or the lumbar intumescence (3–5).

Although PNSTs can develop along any part of a nerve, they most commonly occur in proximity to the nerve root origin, often exhibiting local invasiveness and extension into the vertebral canal. The most frequently reported clinical signs include unilateral limb lameness and muscle atrophy (3, 6, 7).

Reports of distal peripheral nerve sheath tumours (PNSTs) in dogs are limited (8–10). In contrast, human studies show that tumours located at the foot and ankle represent 10.2% (14/137) of all PNSTs, with 14.3% (2/14) of these cases being malignant. Benign schwannomas are the most common tumour type in this region (11). In humans, diagnosis and treatment are often delayed, frequently due to misdiagnosis as tarsal tunnel syndrome or other similar conditions (12, 13).

Amongst canine populations, malignant peripheral nerve sheath tumours (MPNSTs) are more common compared to benign variants. These typically exhibit varied immunoreactivity, with strong expression of Claudin-1, vimentin, S-100, nerve growth factor receptor (NGFR), and SOX10 (14, 15).

SOX10 is a nuclear transcription factor implicated in neural crest differentiation (16, 17). SOX10 reactivity on immunohistochemistry has been found to be more specific for MPNSTs than S-100 and is a sensitive and specific marker for Schwan cell and melanocytic tumours (18). In humans, 67% of MPNSTs are SOX10 positive, with the majority being strongly positive (19). A similar percentage is seen in dogs (66.7%); however, staining is much more variable, with only an epithelioid-variant MPNST and one other tumour variant that showed strong SOX10 labeling (15).

Epithelioid malignant peripheral nerve sheath tumour (EMPNST) is a rare variant of MPNST, accounting for about 5% of these neoplasms. Limited reports exist in veterinary medicine; however, Jo & Fletcher (20) reported clinicopathological features in humans, with 48% of tumours occurring on the lower extremity and only 19% associated with a peripheral nerve; local recurrence (14.3%), metastasis (7.9%), and disease-related death (6.3%) were all relatively uncommon. Strong and diffuse reactivity to S-100 and SOX10 is typical of EMPNST, both in dogs and humans (15, 20–23). However, negative immunolabelling to both S-100 and SOX10 has been previously reported (24).

Neurectomy and amputation, with or without laminectomy, remains the preferred treatment option for peripheral nerve-associated neoplasms, especially for those located more proximally (3, 4, 7, 25). Recurrence rates following amputation typically range from 44% to 78%, with median survival times reported between 5 and 10.5 months (3, 4, 7, 25, 26). Limb-sparing partial neurectomy has been documented as an effective approach for distally located thoracic limb PNSTs, both malignant and benign (8, 9). van Stee et al. (10) reported successful partial neurectomy in 16 dogs, with good to excellent limb function in the majority of the cases and a median survival time of 1,303 days

This case report details an atypical presentation of a distally located EMPNST affecting the tibial nerve with successful limb-sparing neurectomy, atypical immunohistochemical features, and a favorable long-term outcome.

## Case description

An 8-year-old male neutered Dobermann Pinscher presented for further investigations into a five-month history of left pelvic limb lameness and yelping. No significant investigations were performed at the referring veterinarian, and a course of exercise restriction yielded no improvements.

Gait and postural evaluation demonstrated marked left pelvic limb lameness, with myoclonic jerks consistently elicited following flexion of the left tarsus. Postural reactions were preserved, and spinal reflexes were intact. Vertebral column palpation was unremarkable. Manipulation of the left tarsus revealed focal thickening and swelling of the subcutaneous tissues between the distal tibia and fibula. Muscle condition across all limbs was appropriate, with no palpable atrophy or asymmetry. Orthopaedic examination did not reveal additional abnormalities.

**Figure 1:**
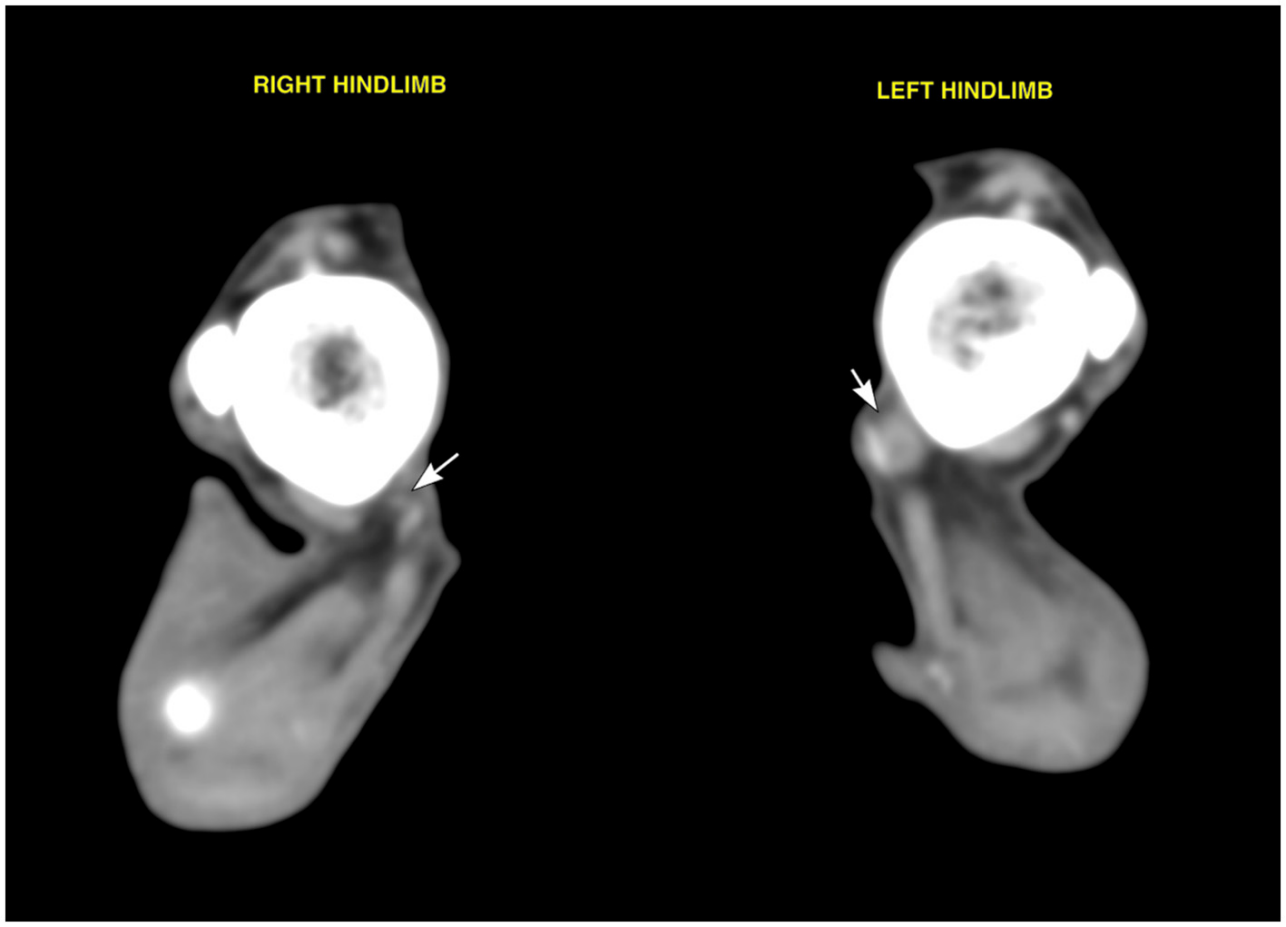
Transverse soft tissue window images of both hindlimbs at the level of the distal third of the tibia showing both tibial nerves (white arrows) running immediately medial to the caudal branch of the saphenous artery. Note the left tibial nerve enlargement when compared to the right side.

**Figure 2:**
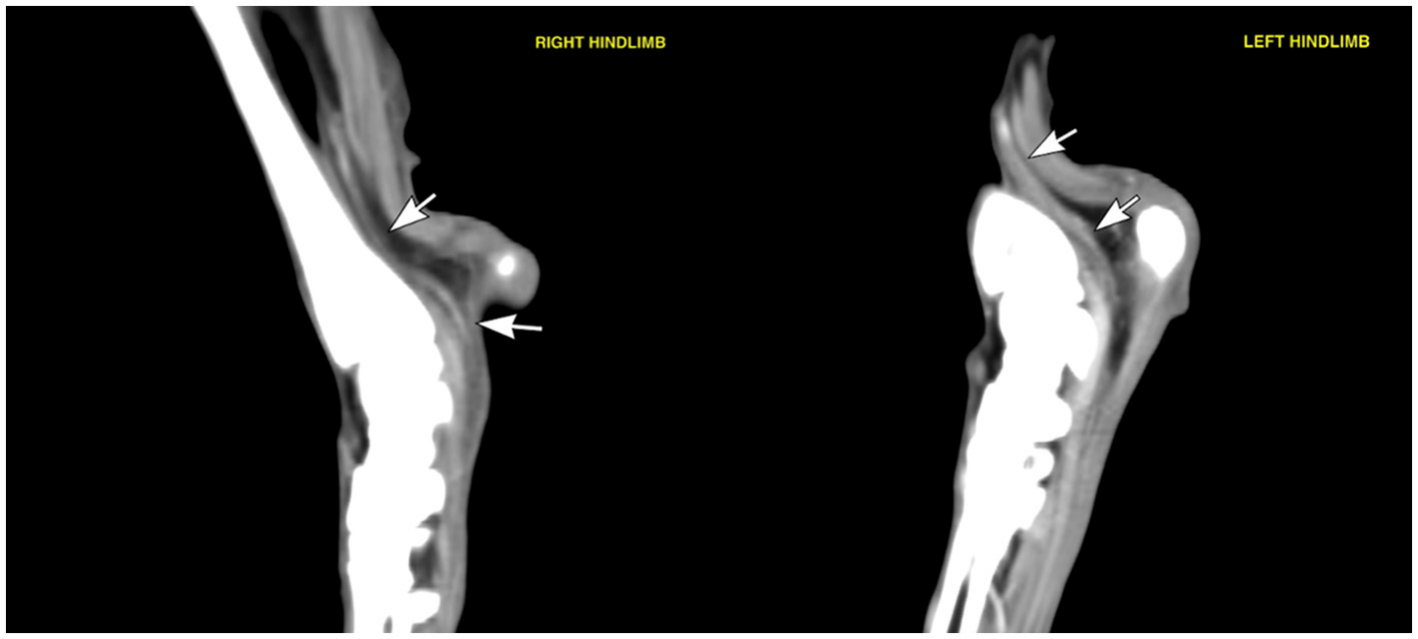
Medial parasagittal soft tissue window images of both hindlimbs showing the tibial nerves (white arrows). Note the enlargement of the left tibial nerve when compared to the right side.

A general anaesthetic was performed for further investigations; electromyography using a Viking Quest (Nicolet biomedical, WI, USA) revealed spontaneous activity with fibrillation potentials and positive sharp waves in the left plantar interosseous muscles, consistent with focal denervation. All other muscle groups in all limbs were otherwise unaffected. Computed tomography (CT) of the tarsi was performed in dorsal recumbency using a 64-slice Siemens SOMATOM Perspective scanner (Siemens Healthcare). The scan parameters included helical acquisition, slice thickness of 0.6 mm, 130 kVp, 160 mAs, and matrix size 512 × 512. The study included unenhanced images and venous post-contrast images following intravenous administration of 2 mL/kg of iodinated non-ionic contrast agent (Omnipaque 300 mg/mL solution for injection, Iohexol, GE Healthcare) using automated injection. The venous series was acquired approximately 75 s after initial injection. The images were displayed using a bone window [window level −500 Hounsfield units (HU), window width 1,400 HU] for the high-frequency reconstruction algorithm, and a soft tissue window (window level 50 HU, window width 350 HU) for the medium-frequency reconstruction algorithm and were evaluated using DICOM viewing software (Osirix, version 7.0.1, Pixmeo). CT revealed a focal thickening of the left tibial nerve at the level of the calcaneal region, extending approximately between the distal third of the tibia to the talocalcaneal joint (Figures 1, 2). The enlarged nerve measured up to 4 mm in thickness and showed moderate homogeneous contrast enhancement. The distal divisions of the tibial nerve (lateral and medial plantar nerves) appeared normal in size and attenuation, being symmetrical when compared to the right hindlimb. Additionally, mild atrophy of the left gastrocnemius and left deep and superficial digital flexor muscles was identified.

**Figure 3:**
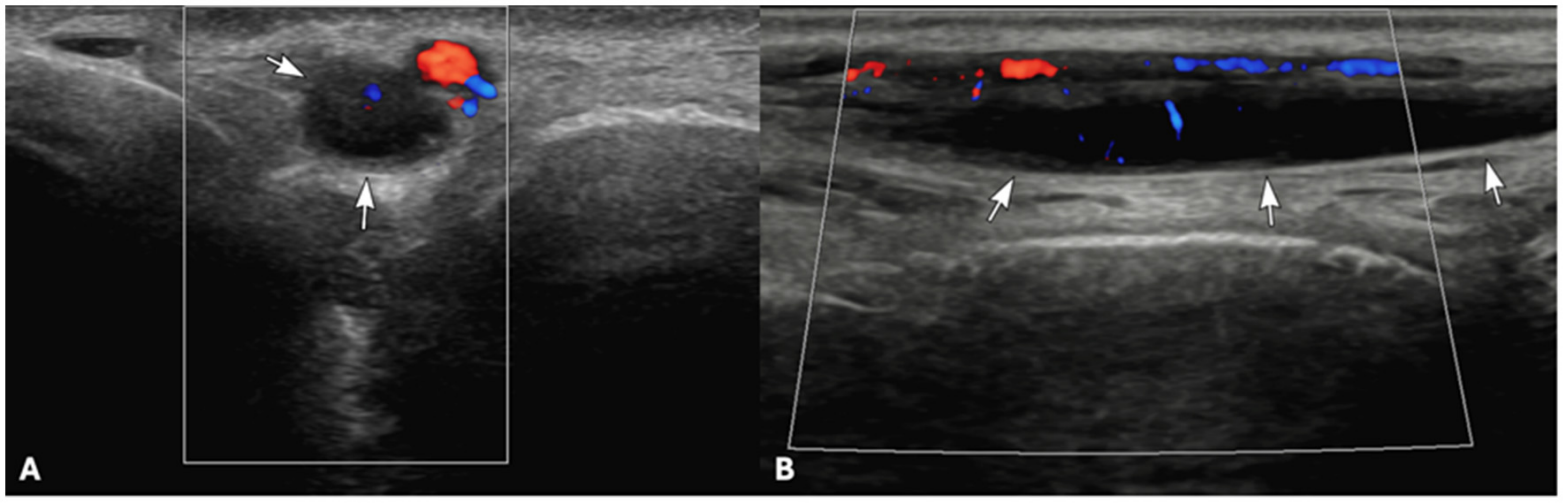
Transverse **(A)** and sagittal sonogram images **(B)** showing the enlarged left tibial nerve. On Doppler interrogation, the caudal branch of the saphenous artery is visible immediately dorsolateral to the enlarged nerve.

Ultrasound examination showed moderate thickening of the left tibial nerve (4 mm in diameter) compared to the right tibial nerve (1 mm in diameter), being otherwise of normal homogenous and hypoechoic appearance (Figure 3). There was no evidence of regional soft tissue changes or popliteal lymphadenomegaly. Ultrasound-guided fine needle aspirates of the left tibial nerve were acquired without immediate complication. Cytological evaluation was indicative of a histiocytic neoplastic process, characterized by a mixed inflammatory cell population predominantly composed of histiocytes exhibiting marked cytologic atypia (data not provided).

Comprehensive staging via full-body computed tomography (CT) was recommended prior to surgical resection to evaluate for potential metastatic dissemination or concurrent lesions. However, this diagnostic step was declined by the owner due to personal considerations, and surgical planning proceeded based on the available imaging and clinical findings. A limb-sparing technique, via partial neurectomy of the left tibial nerve, was performed, with a wide proximodistal resection to ensure complete removal of the mass and clean margins. An elliptical skin incision, approximately 5–6 mm wide, was made directly over the thickened nerve, on the medial aspect of the distal crural area, in the space between the common calcaneal tendon and the caudal aspect of the tibia. The incision was extended distally following the nerve to the level of the proximal aspect of the metatarsal bones. The nerve was circumferentially dissected from its surrounding connective tissue so that an en bloc resection was attempted, maintaining a rim of healthy connective tissue around the thickened nerve. Surgical dissection was performed in a proximal-to-distal direction. The tibial nerve was initially transected approximately 2.5 cm proximal to the cranial margin of the observed thickening. The caudal cutaneous sural nerve was subsequently transected at its point of convergence with the tibial nerve.

The nerve was transected sharply, 2–3 cm distal to the visible extent of the swelling, immediately proximal to the bifurcation into medial and lateral plantar nerves, on the medial aspect of the calcaneus. Wound closure was routine, using 3/0 PDS Plus^®^ (Ethicon^®^) in a simple continuous pattern for the subcutaneous tissue, 3/0 Monocryl Plus^®^ (Ethicon^®^) for a continuous intradermal, and Dermabond^®^ skin adhesive for the final skin closure. The popliteal lymph node was removed via a separate incision on the caudal aspect of the stifle joint. Closure was also routine.

During the post-operative recovery, a marked swelling was evident at the level of the left hock, and following self-traumatisation, the distal portion of the surgical site reopened. This was allowed to heal by secondary intention, with no further complications seen. At reassessment two weeks after surgery, minimal lameness was evident in the left pelvic limb.

Numerous transverse and longitudinal sections of the left tibial nerve mass samples fixed in 10% buffered formalin solution were paraffin-embedded in tissue cassettes, and 4 μm thick sections were cut using a microtome and placed on a microscope slide. Slides were then stained with Haematoxylin and Eosin using an automated histochemical stainer and coverslipped. Effacing and replacing the endoneurium and infiltrating the perineurium is an unencapsulated, poorly demarcated, densely cellular, malignant neoplasm. Diffuse sheets of neoplastic polygonal cells occasionally engulf pre-existing, degenerating axons. Individual cells are round with distinct borders, a small amount of eosinophilic granular cytoplasm, and a single round to ovoid nucleus with finely stippled basophilic chromatin and occasionally one large prominent nucleolus. Anisocytosis and anisokaryosis are moderate, and six mitotic figures are observed in 2.37 mm^2^. Within the surrounding epineurium, there is multifocal, moderate, lymphoplasmacytic perivascular cuffing. There was no evidence of epithelial structures, melanin pigment, or lymphovascular invasion, and so differentials of a metastatic lesion, carcinoma, or melanoma were considered unlikely. IHC with antibodies against multicytokeratin (epithelial cell marker) and MelanA and PNL2 (melanocyte markers) was not performed due to financial constraints and sample availability.

**Figure 4:**
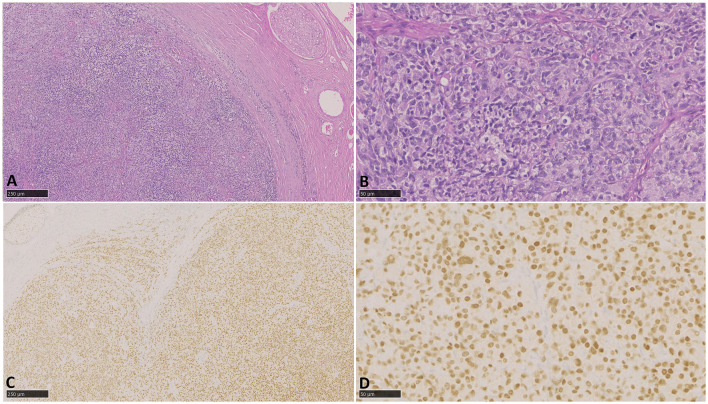
Photomicrographs of an epithelioid malignant nerve sheath tumour. **(A, B)** H&E, 40x−200x, Effacing the endoneurium and infiltrating the perineurium is a malignant neoplasm arranged in diffuse sheets of polygonal cells with a small amount of eosinophilic granular cytoplasm and moderate pleomorphism. **(C, D)** Immunohistochemistry, Sox10, 40x−200x, Neoplastic cells exhibit diffuse strong nuclear immunolabelling with antibodies against Sox10.

Additional 4 μm thick unstained sections of the formalin-fixed paraffin-embedded blocks were cut using a microtome and placed on a microscope slide. Slides were run through a Leica Bond MAX automated stainer using the DS9800 BOND Polymer Refine Detection System and the Leica Buffer ready to use dilute (1:10 tris buffered saline) and ready to use antigen retrieval 1 (pH 6) or 2 (pH 9) (Leica Biosystems, Wetzlar, Germany). Using appropriate epitope retrieval buffers, sections were deparaffinised and rehydrated, followed by heat-induced epitope retrieval and incubation with the primary antibody according to optimized conditions. Detection used a polymer-based system with DAB chromogen, and slides were counterstained with haematoxylin and cover-slipped. Appropriate positive and negative controls were included in each run. Neoplastic cells exhibit diffuse strong nuclear immunolabelling with antibodies against Sox10 and negative immunolabelling with antibodies against S100, CD3, CD20, and IBA-1 (Figure 4).

At 11-month postoperative follow-up, the patient exhibited no signs of pain or lameness in the previously affected left pelvic limb. However, the owner reported recent-onset scuffing of the right thoracic and pelvic limbs, with noticeable nail wear consistent with abnormal limb placement or proprioceptive deficits. Neurological examination revealed normal postural reactions and intact spinal reflexes in all limbs. To further evaluate the reported gait abnormalities, a pressure-sensitive gait analysis was conducted. This consistently demonstrated right-sided gait disturbances, characterized by audible nail scuffing, suggesting possible upper motor neuron involvement or cervical spinal pathology.

CT of the tarsi was repeated using similar parameters to those previously described. The left tibial nerve was not clearly identified due to the presence of ill-defined, soft tissue non-contrast enhancing attenuation within the subcutaneous tissues of the surgical site. The cervical spine, thorax and abdomen were also included in the study. The patient was positioned in sternal recumbency, and the scan parameters were the following: helical acquisition, slice thickness of 3 mm, 130 kVp, 180 mAs and matrix size 512 × 512. The series included unenhanced images and venous post-contrast images following intravenous administration of 2 mL/kg of iodinated non-ionic contrast agent (Omnipaque 300 mg/mL solution for injection, Iohexol, GE Healthcare) using automated injection. The abdominal series also included arterial post-contrast images obtained at approximately 15 seconds using an automatic bolus tracking technique. CT of the cervical spine revealed misalignment between the C5 and C6 vertebrae due to craniodorsal tilting of the vertebral bodies with associated narrowing of the intervertebral disc space and dorsal protrusion of the intervertebral disc into the ventral aspect of the vertebral canal. These findings result in moderate narrowing of the vertebral canal, which was indicative of disc-associated cervical spondylomyelopathy (CSM), a condition that could account for the observed gait abnormalities. Additional findings included multiple splenic nodules of undetermined significance, generalized bronchial wall thickening suggestive of chronic bronchitis, and mild spondylosis deformans at the L7-S1 vertebral junction.

Ultrasound examination identified the tibial nerve, which was focally thickened at the distal end (measuring approximately 3.5 mm in diameter) when compared with the more proximal portion of this nerve (measuring 1–2mm in diameter). Ill-defined, hyperechoic tissue was visible surrounding the distal end of the nerve and extending distally along the regional subcutaneous tissues. Ultrasound-guided fine needle aspirates of this tissue swelling were taken, although this was poorly cellular and non-diagnostic. A post-operative fibrosis was considered most likely, with tumour recurrence considered unlikely. Further diagnostic workup, including MRI and cerebrospinal fluid analysis, as well as potential surgical stabilization of the C5–C6 segment, was discussed with the owner. However, these recommendations were ultimately declined.

At 24 months follow-up, no recurrence of pain or functional impairment was observed in the left pelvic limb, and repeat ultrasonography of the prior transection site showed resolution of the previously noted focal thickening. Meanwhile, the patient exhibited progression of right-sided tetraparesis, characterized by increased scuffing and delayed proprioception in the right thoracic and pelvic limbs, consistent with worsening cervical spinal disease. The patient was unfortunately euthanised three months later following an acute gastric dilatation volvulus.

## Discussion

This case represents the first report of a distally located epithelioid malignant PNST in a dog; successful limb-sparing neurectomy was performed and a good long-term outcome was achieved. Corresponding with previous studies (7, 9, 27), the time to diagnosis was prolonged due to the initial subtlety of the clinical signs. Whilst a palpable and painful mass can be a common finding on examination (28, 29), in this case, a mass was only identified after the region was shaved prior to ultrasound.

Multiple techniques have been described for the diagnosis of PNST, including computed tomography (CT), electromyography, magnetic resonance imaging (MRI), and ultrasound (6, 30–32). In this case, CT was used initially alongside a combination of electrodiagnostics and ultrasonography, which proved beneficial in identifying a neuropathy and the presence of a solitary mass lesion affecting the tibial nerve.

Although metastases affecting the liver, kidneys, lungs, and lymph nodes have been documented in previous studies involving epithelioid MNSTs (23), cross-sectional imaging of the thorax and abdomen—such as computed tomography (CT)—was not performed at the time of diagnosis in this case due to financial considerations. As a result, the presence of subclinical metastatic disease at presentation could not be definitively excluded at the time of surgery. CT of the tarsi, thorax, and abdomen, before and after contrast administration, was performed at a follow-up 12 months later, with no evidence of local recurrence or metastasis. Repeat ultrasound of the left tibial region at 24 months was similarly unremarkable. Nevertheless, the possibility of occult metastasis should not be overlooked, particularly in malignant subtypes such as epithelioid or anaplastic variants. Comprehensive staging—including thoracic and abdominal imaging—is therefore advisable when clinical suspicion exists or when definitive therapeutic planning is being considered. In our case, the lack of staging was a severe limitation that was linked to financial restrictions.

The decision to pursue a limb-sparing neurectomy in this case was based on the anatomical location of the affected segment of the tibial nerve, which primarily innervates the gastrocnemius, popliteus, and the superficial and deep digital flexor muscles (33). Given the specific motor function of these muscles and their limited contribution to weight-bearing or primary limb advancement during gait, significant postoperative gait abnormalities were not anticipated. Two cases of tibial nerve partial neurectomy were reported by van Stee et al. (10) as part of a case series involving 16 dogs with limb-sparing surgery, both of which showed good improvement of a limb, with survival times of 2,227 and 4,639 days. Unfortunately, our case was euthanised for reasons unrelated to his neoplasia; our post-operative findings were similar to those of van Stee et al. (10), with no evidence of long-term limb dysfunction.

Achieving a histopathological diagnosis in this case was challenging. Based on the cytological appearance of the neoplasm, a lymphocytic or histiocytic origin was initially suspected; however, negative labeling was observed with the CD3, CD20, and IBA-1 markers. The majority of published literature suggests a strong reactivity to both SOX10 and S-100 with EMPNST in humans (14, 18, 20–22, 34). No published literature on EMPNST in dogs has previously evaluated both SOX10 and S-100 together; Tekavec et al. (15) reported a strong reactivity to SOX10 in a single case, whilst Garcia et al. (23) reported reactivity to S-100. Our report is novel in reporting both S-100 and SOX10 immunoreactivity; however, it is unusual in nature, given the strong reactivity to SOX10 but negative labeling with S-100. The absence of immunoreactivity for S-100 may be due to poor differentiation of the neoplasm, so neoplastic cells do not express antigens associated with S-100 immunolabelling. No other positive immunohistochemistry markers have been reported with EMPNSTs in veterinary literature (15, 23, 35). The combination of morphological characteristics and SOX10 strong reactivity was conclusive for an epithelioid malignant peripheral nerve sheath tumour.

## Conclusion

To the author's knowledge, this represents the first report of a hindlimb EMPNST, with successful management using limb-sparing partial neurectomy and demonstrating a good long-term outcome. Similarly, this is the first report in dogs of EMPNST where both S-100 and SOX10 immunohistochemistry was performed, with a novel finding of S-100 negativity.

Achieving a histopathological diagnosis was challenging, and a combination of both SOX10 and S-100 immunohistochemistry should be considered in cases where EMPNST is suspected.

## Data Availability

The raw data supporting the conclusions of this article will be made available by the authors, without undue reservation.

## References

[1] Grüntzig K Graf R Hässig M Welle M Meier D Lott G . The Swiss canine cancer registry: a retrospective study on the occurrence of tumours in dogs in Switzerland from 1955 to 2008. J Comp Pathol. (2015) 152:161–71. doi: 10.1016/j.jcpa.2015.02.005 25824119

[2] Erlandson RA Woodruff JM. Peripheral nerve sheath tumors: an electron microscopic study of 43 cases. Cancer. (1982) 49:273–87. doi: 10.1002/1097-0142(19820115)49:2&lt;273::AID-CNCR2820490213&gt;3.0.CO;2-R 7053827

[3] Cooper-Khan RS Frankovich AN Thompson CA Thomovsky SA Lewis MJ. Clinical findings and outcome in 30 dogs with presumptive or confirmed nerve sheath tumors. Vet Sci. (2024) 11:192. doi: 10.3390/vetsci11050192 38787164 PMC11125868

[4] Potamopoulou M Petite A Findji L. Combined forequarter amputation and hemilaminectomy for treatment of canine peripheral nerve sheath tumors of the brachial plexus invading the spinal canal: surgical technique and outcome in nine dogs. Vet Surg. (2024) 53:1477–84. doi: 10.1111/vsu.14166 39315668

[5] Swift KE McGrath S Nolan MW Young M Reese M Rao S . Clinical and imaging findings, treatments, and outcomes in 27 dogs with imaging diagnosed trigeminal nerve sheath tumors: a multi-center study. Vet Radiol Ultrasound. (2017) 58:679–89. doi: 10.1111/vru.12535 28758278

[6] le Chevoir M Thibaud JL Labruyère J Uriarte A De Fornel-Thibaud P Moissonnier P . Electrophysiological features in dogs with peripheral nerve sheath tumors: 51 cases (1993–2010). J Am Vet Med Assoc. (2012) 241:1194–201. doi: 10.2460/javma.241.9.1194 23078567

[7] Brehm D Vite C Steinberg H Haviland J van Winkle T. A retrospective evaluation of 51 cases of peripheral nerve sheath tumors in the dog. J Am Anim Hosp Assoc. (1995) 31:349–59. doi: 10.5326/15473317-31-4-349 7552669

[8] Miyaho N Mochizuki M Honnami M. Forelimb lameness caused by malignant peripheral nerve sheath tumors of the median nerve in a dog: a case report. J Vet Med Sci. (2024) 86:24–38. doi: 10.1292/jvms.24-0038 38945917 PMC11300132

[9] Salmina AG Castelli E Beckmann K Mauri N. Challenges in diagnosing a peripheral nerve sheath tumor of the ulnar nerve in a dog – a case report. Schweiz Arch Tierheilkd. (2022) 164:265–71. doi: 10.17236/sat00349 35232717

[10] van Stee L Boston S Teske E Meij B. Compartmental resection of peripheral nerve tumours with limb preservation in 16 dogs (1995–2011). Vet J. (2017) 226:40–5. doi: 10.1016/j.tvjl.2017.07.002 28911840

[11] Carvajal JA Cuartas E Qadir R Levi AD Temple HT. Peripheral nerve sheath tumors of the foot and ankle. Foot Ankle Int. (2011) 32:163–7. doi: 10.3113/FAI.2011.0163 21288416

[12] Milnes HL Pavier JC. Schwannoma of the tibial nerve sheath as a cause of tarsal tunnel syndrome—a case study. The Foot. (2012) 22:243–6. doi: 10.1016/j.foot.2012.03.005 22560193

[13] Wiles CM Whitehead S Ward AB Fletcher CD. Not tarsal tunnel syndrome: a malignant “Triton” tumour of the tibial nerve. J Neurol Neurosurg Psychiatry. (1987) 50:479–81. doi: 10.1136/jnnp.50.4.479 3585361 PMC1031887

[14] Chijiwa K Uchida K Tateyama S. Immunohistochemical evaluation of canine peripheral nerve sheath tumors and other soft tissue sarcomas. Vet Pathol. (2004) 41:307–18. doi: 10.1354/vp.41-4-307 15232130

[15] Tekavec K Švara T Knific T Gombač M Cantile C. Histopathological and immunohistochemical evaluation of canine nerve sheath tumors and proposal for an updated classification. Vet Sci. (2022) 9:204. doi: 10.3390/vetsci9050204 35622732 PMC9144584

[16] Kuhlbrodt K Herbarth B Sock E Hermans-Borgmeyer I Wegner M. Sox10, a novel transcriptional modulator in glial cells. J Neurosci. (1998) 18:237–50. doi: 10.1523/JNEUROSCI.18-01-00237.1998 9412504 PMC6793382

[17] Britsch S Goerich DE Riethmacher D Peirano RI Rossner M Nave KA . The transcription factor Sox10 is a key regulator of peripheral glial development. Genes Dev. (2001) 15:66–78. doi: 10.1101/gad.186601 11156606 PMC312607

[18] Karamchandani JR Nielsen TO van de Rijn M West RB. Sox10 and S100 in the diagnosis of soft-tissue neoplasms. Appl Immunohistochem Mol Morphol. (2012) 20:445–50. doi: 10.1097/PAI.0b013e318244ff4b 22495377

[19] Kang Y Pekmezci M Folpe AL Ersen A Horvai AE. Diagnostic utility of SOX10 to distinguish malignant peripheral nerve sheath tumor from synovial sarcoma, including intraneural synovial sarcoma. Mod Pathol. (2014) 27:55–61. doi: 10.1038/modpathol.2013.115 23929265

[20] Jo VY Fletcher CDM. Epithelioid malignant peripheral nerve sheath tumor. Am J Surg Pathol. (2015) 39:673–82. doi: 10.1097/PAS.0000000000000379 25602794

[21] Heatley N Kolson Kokohaare E Strauss DC Hallin M Jones RL Fisher C . Epithelioid malignant peripheral nerve sheath tumor arising in schwannoma. Rare Tumors. (2020) 12:2036361320950862. doi: 10.1177/2036361320950862 32913618 PMC7443986

[22] Alwhabi MK Almalki MA Alrumeh AS Almadan NM Alfaifi SA. Epithelioid malignant peripheral nerve sheath tumor: a rare tumor with an unusual presentation in the ankle: a case report and literature review. J Musculoskelet Surg Res. (2022) 6:288. doi: 10.25259/JMSR_95_2022

[23] Garcia P Sánchez B Sánchez MA González M Rollán E Flores JM. Epithelioid malignant peripheral nerve sheath tumour in a dog. J Comp Pathol. (2004) 131:87–91. doi: 10.1016/j.jcpa.2003.12.012 15144803

[24] Yamashita K Funauchi Y Hayakawa K Ae K Matsumoto S Ikuta K . S100-negative epithelioid malignant peripheral nerve sheath tumor with possible perineurial differentiation. Virchows Archiv. (2022) 480:1269–75. doi: 10.1007/s00428-021-03218-y 34635937

[25] Stokes R Wustefeld-Janssens BG Hinson W Wiener DJ Hollenbeck D Bertran J . Surgical and oncologic outcomes in dogs with malignant peripheral nerve sheath tumours arising from the brachial or lumbosacral plexus. Vet Comp Oncol. (2023) 21:739–47. doi: 10.1111/vco.12938 37727977

[26] Lacassagne K Hearon K Berg J Séguin B Hoyt L Byer B . Canine spinal meningiomas and nerve sheath tumours in 34 dogs (2008–2016): distribution and long-term outcome based upon histopathology and treatment modality. Vet Comp Oncol. (2018) 16:344–51. doi: 10.1111/vco.12385 29363264

[27] Anderson O Langley-Hobbs SJ. A peripheral nerve sheath tumour in the median nerve of a dog. Vet Rec Case Rep. (2022) 10:e323. doi: 10.1002/vrc2.323

[28] Targett MP Dyce J Houlton JEF. Tumours involving the nerve sheaths of the forelimb in dogs. J Small Anim Pract. (1993) 34:221–5. doi: 10.1111/j.1748-5827.1993.tb02669.x

[29] Carmichael S Griffiths I. Tumours involving the brachial plexus in seven dogs. Vet Rec. (1981) 108:435–7. doi: 10.1136/vr.108.20.435 7292911

[30] Kraft S Ehrhart EJ Gall D Klopp L Gavin P Tucker R . Magnetic resonance imaging characteristics of peripheral nerve sheath tumors of the canine brachial plexus in 18 dogs. Vet Radiol Ultrasound. (2007) 48:1–7. doi: 10.1111/j.1740-8261.2007.00195.x 17236352

[31] Guilherme S Benigni L. Ultrasonographic anatomy of the brachial plexus and major nerves of the canine thoracic limb. Vet Radiol Ultrasound. (2008) 49:577–83. doi: 10.1111/j.1740-8261.2008.00424.x 19051650

[32] Niles JD Dyce J Mattoon JS. Computed tomography for the diagnosis of a lumbosacral nerve sheath tumour and management by hemipelvectomy. J Small Anim Pract. (2001) 42:248–52. doi: 10.1111/j.1748-5827.2001.tb02030.x 11380019

[33] de Lahunta A Glass E Kent M. de Lahunta's Veterinary Neuroanatomy and Clinical Neurology. Philadelphia: Elsevier - OCHE (2020). p. 110.

[34] Laskin WB Weiss SW Bratthauer GL. Epithelioid variant of malignant peripheral nerve sheath tumor (malignant epithelioid schwannoma). Am J Surg Pathol. (1991) 15:1136–45. doi: 10.1097/00000478-199112000-00004 1746681

[35] Pumarola M Añor S Borràs D Ferrer I. Malignant epithelioid schwannoma affecting the trigeminal nerve of a dog. Vet Pathol. (1996) 33:434–6. doi: 10.1177/030098589603300411 8817843

